# Accurate interpretation of genetic variants in sudden unexpected death in infancy by trio-targeted gene-sequencing panel analysis

**DOI:** 10.1038/s41598-021-00962-8

**Published:** 2021-11-02

**Authors:** Keita Shingu, Takehiko Murase, Takuma Yamamoto, Yuki Abe, Yoriko Shinba, Masahide Mitsuma, Takahiro Umehara, Hiromi Yamashita, Kazuya Ikematsu

**Affiliations:** 1 Division of Forensic Pathology and Science, Department of Medical and Dental Sciences, Graduate School of Biomedical Sciences, School of Medicine, Nagasaki University, Nagasaki, Japan; 2 Departments of Pediatrics, Nagasaki University Graduate School of Biomedical Sciences, Nagasaki, Japan; 3 Division of Forensic Dental Science, Department of Medical and Dental Sciences, Graduate School of Biomedical Sciences, School of Medicine, Nagasaki University, Nagasaki, Japan; 4 Present Address: Department of Legal Medicine, Hyogo College of Medicine, 1-1 Mukogawa-cho, Nishinomiya, Hyogo 663-8501 Japan

**Keywords:** Disease genetics, Paediatrics

## Abstract

In sudden unexpected death in infancy cases, postmortem genetic analysis with next-generation sequencing potentially can extract candidate genes associated with sudden death. However, it is difficult to accurately interpret the clinically significant genetic variants. The study aim was to conduct trio analysis of cases of sudden unexpected death in infancy and their parents to more accurately interpret the clinically significant disease-associated gene variants associated with cause of death. From the TruSight One panel targeting 4813 genes we extracted candidate genetic variants of 66 arrhythmia-, 63 inherited metabolic disease-, 81 mitochondrial disease-, and 6 salt-losing tubulopathy-related genes in 7 cases and determined if they were de novo or parental-derived variants. Thirty-four parental-derived variants and no de novo variants were found, but none appeared to be related to the cause of death. Using trio analysis and an in silico algorithm to analyze all 4813 genes, we identified *OBSCN* of compound heterozygous and *HCCS* of hemizygous variants as new candidate genetic variants related to cause of death. Genetic analysis of these deceased infants and their living parents can provide more accurate interpretation of the clinically significant genetic variants than previously possible and help confirm the cause of death.

## Introduction

Sudden unexpected death in infancy (SUDI) is a term that has been variably used to refer to all cases of sudden and unexpected deaths in infancy, including sudden infant death syndrome (SIDS)^1^. In 2015, SIDS was the third leading cause of death at age 0 in Japan, with a mortality rate of 9.5 per 100,000 population^2^. However, SUDI is thought to be more common because of the difficult distinction between SIDS and accidental asphyxia or natural diseases, such as arrhythmias and inherited metabolic disease^2^. Arrhythmia, inherited metabolic disease, mitochondrial disease, and salt-losing tubulopathy are linked to sudden unexpected death (SUD)^3–13^. Thus, SUDI is considered to be the severest form of various related diseases. The standard methods of determining the cause of death in SUDI cases include conventional autopsy (macroscopic autopsy and detailed microscopic examination^14^), toxicology, biochemical tests, and recently, metabolic autopsy^15^. However, in most SUDI cases, these methods do not reveal the exact cause of death, hence the urgent need to develop novel diagnostic methods.

Recently, some studies have shown that genetic analysis using next-generation sequencing (NGS) of SUDI cases was effective in diagnosing arrhythmias and inherited metabolic disease ^9,10^. On the other hand, it has been reported that some of the variants in arrhythmia-related genes found in SUD cases were not associated with cause of death^16,17^. In other words, an accurate interpretation of the association between variants in disease-related genes found in SUD cases and sudden death is important in determining cause of death. However, even if variants in arrhythmia-related genes are found, it is difficult to obtain findings that can be judged as arrhythmia from macroscopic, pathological, and biochemical examinations. Most conventional NGS studies were limited to genetic analysis of sudden death cases only. Recently, familial gene analysis of cardiac sudden death cases and their parents was shown to be effective for diagnosis of cause of death^18–25^. However, most family analyses have targeted genes for arrhythmia, so it would be useful to target not only those genes but also any other disease-related genes. Furthermore, few studies have reported family genetic analyses with NGS that focused on SUDI. Since SUDI is considered to be the severest form of various diseases, it would be useful to analyze the genes associated not only with arrhythmia but also with other diseases that can cause sudden death. Therefore, we thought that genetic analysis of SUDI cases and their parents would lead to a more accurate interpretation of the clinically significant genetic variants associated with cause of death.

The study aim was to perform trio analysis of SUD infants and their parents to interpret the clinically significant disease-associated gene variants associated with the cause of death more accurately. In addition, we used trio analysis to extract novel candidate gene variants and examined whether these extracted variants were associated with cause of death.

## Results

### Case collection

**Figure 1:**
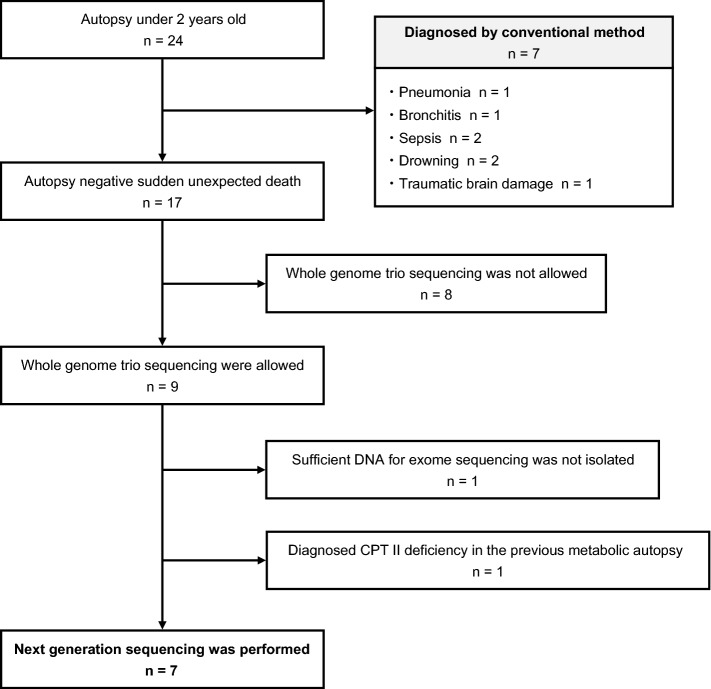
Flowchart of the selection process showing the number of participants in this study. Autopsy negative means that the case was not diagnosed even after macroscopic and microscopic examinations and a toxicology examination. Abbreviation: CPT II: carnitine palmitoyltransferase II.

Among the 24 autopsied cases, 17 could not be diagnosed using conventional methods. From the 17 undiagnosed cases, 9 patients’ parents gave consent for whole-genome sequencing. One of the nine cases was diagnosed with carnitine palmitoyltransferase II deficiency by metabolic autopsy^26^. In another case, we could not extract a sufficient amount of DNA for targeted gene-sequencing panel analysis. A total of seven SUDI cases (two males, five females; age range: 3 months–2 years) were selected (Fig. 1). The case 2 parents had a consanguineous marriage (Table 1).

**Table 1:** Summary of SUDI cases and their parents and results of sequencing analysis.

Trio No	Subject	Age	Sex	Total aligned reads	Total aligned bases	Targeted aligned reads	Mean region coverage depth	Target coverage at 20X (%)
**1**	Case	3 M	Female	12,491,263	1,494,300,281	10,542,312	85	82
	Father	–	–	21,352,420	3,069,189,175	14,139,830	124.2	96.9
	Mother	–	–	15,157,248	2,148,307,197	10,561,623	91.9	94.1
**2**	Case	1Y8M	Female	14,318,515	1,727,573,727	10,812,331	86.6	88.4
	Father	–	–	18,296,664	2,543,663,623	12,035,904	101.2	96.4
	Mother	–	–	20,837,322	2,915,885,754	13,477,515	113.9	95.5
**3**	Case	3 M	Female	14,482,070	2,037,650,595	9,811,216	84.8	93.8
	Father	–	–	17,437,111	2,451,099,641	11,825,077	101.7	95.6
	Mother	–	–	17,211,772	2,432,468,086	11,476,012	99.2	95.1
**4**	Case	3 M	Female	20,731,617	2,981,575,844	14,700,318	129.4	97
	Father	–	–	17,000,730	2,364,257,419	12,397,912	106.9	95.7
	Mother	–	–	14,574,427	2,070,845,131	10,579,807	92.6	94.7
**5**	Case	4 M	Female	16,762,916	2,344,786,584	10,088,104	85.6	95.3
	Father	–	–	17,644,526	2,424,775,119	11,695,666	100.4	92.1
	Mother	–	–	22,127,138	2,971,368,392	15,889,907	134.2	95.4
**6**	Case	2Y	Male	16,932,684	2,353,138,678	10,368,292	87.6	95.8
	Father	–	–	15,497,810	2,083,213,340	10,775,295	92.5	79.7
	Mother	–	–	23,474,057	3,265,426,029	16,050,903	136.9	96.8
**7**	Case	6 M	Male	18,961,926	2,635,964,881	11,983,786	100.7	96.2
	Father	–	–	19,247,261	2,676,991,782	12,048,382	101.9	95.5
	Mother	–	–	14,468,867	2,009,952,449	8,700,560	75.2	80.2

Trio 2 parents had a consanguineous marriage.

### Target sequence of sudden death cases

On average, ~ 17.5 million total reads were produced and ~ 11.9 million reads mapped to the targeted region in each sample. The mean coverage of the coding sequence was 101.5 ± 33.7 reads, with an overall average gene level coverage ≥ 20 reads of 93.0% ± 10.7%. After the filtering steps, 1897 variants in the 4813 target genes were identified, which corresponded to an average of 271 variants per case.

### Detected variants aligned to the 216 target genes

**Figure 2:**
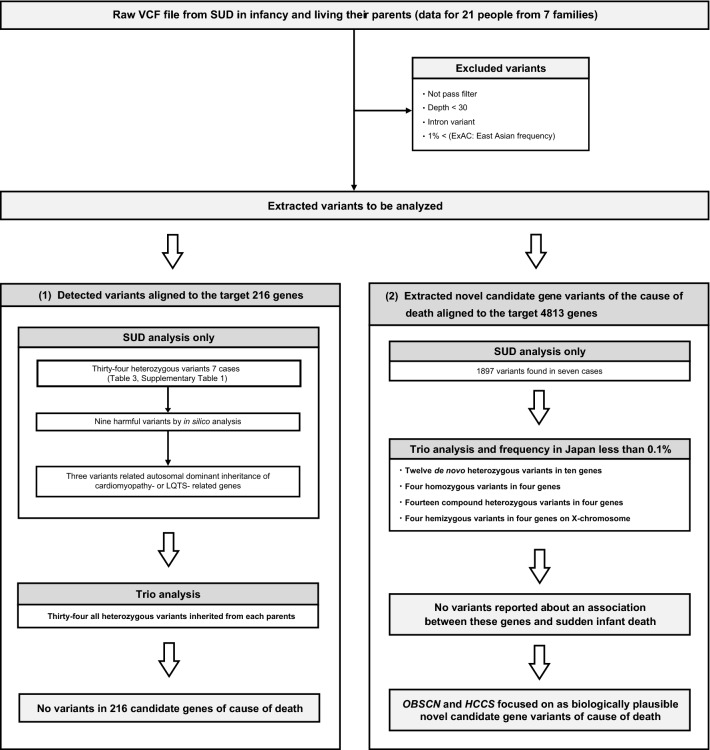
Flowchart of the selection process showing candidate genes for cause of death in this study. The frequency of variants in Japanese people was based on ToMMo and HGVD data. (**a**) The target 216 genes are listed in Table 2. (**b**) All genes included in the TruSight One panel are shown in Supplementary Text 1. Abbreviations: SUD: sudden unexpected death.

Thirty-four (18 non-synonymous and 16 synonymous) detected variants were aligned with the 216 target genes. Eleven arrhythmia-, two cardiomyopathy-, three metabolic disease-, fifteen mitochondrial disease-, and three salt-losing tubulopathy-related genes were included. All of these genetic variants were heterozygous and did not contain any de novo gene variants (Tables 2 and 3, Supplementary Table 1, and Fig. 2).

**Table 2:** Arrhythmia-, inherited metabolic disease-, mitochondrial disease-, and salt-losing tubulopathy-related genes investigated in this study.

**Arrhythmia**	Brugada syndrome	*SCN5A, GPD1L, CACNA1C, CACNB2, SCN1B, KCNE3, SCN3B, KCNH2, KCNJ8, CACN2D1, RANGRF, KCNE5, KCND3, HCN4, SLMAP, TRPM4, SCN2B, FGF12, SCN10A*
	Long QT syndrome	*KCNQ1, KCNH2, SCN5A, ANK2, KCNE1, KCNE2, KCNJ2, CACNA1C, CAV3, SCN4B, AKAP9, SNTA1, KCNJ5, CALM1, CALM2*
	Short QT syndrome	*KCNH2, KCNQ1, KCNJ2, CACNA1C, CACNB2, CACN2D1*
	PCCD	*SCN5A, LMNA, EMD, SCN1B, TRPM4, GJA5*
	CPVT	*RYR2, CASQ2, TRDN, CALM1, CALM2*
	ARVC	*PKP2, DSC2, DSG2, JUP, DSP*
	Others	*CAMK2D, CALM3, DES, DPP6, GJA1, GJC1, KCNA5, KCNE4, KCNIP2, KCNJ3, KCNK17, KCNN2, MYBPC3, MYH6, MYH7, NCS1, NKX2-5, NPPA, PLN, SLC8A1, TAZ, TBX3TBX5, TCAP*
**Inherited metabolic disease**	Organic acid	*ACAT1, AUH, ETFA, ETFB, ETFDH, GCDH, HLCS, HMGCL, HMGCS2, IVD, LMBRD1, MCCC1, MCCC2, MMAA, MMAB, MMACHC, MMADHC, MUT, OPA3, PCCA, PCCB*
	Amino acid	*ASL, ASS1, BCKDHA, BCKDHB, BCKDK, CBS, CPS1, DLD, DBT, MAT1A, NAGS, OTC, PAH, PCBD1, PTS, QDPR, SLC25A13*
	Fatty acid oxidation	*ACADM, ACADVL, CPT1A, CPT2, HADHA, HADHB, SLC22A5, SLC25A20*
	Carbohydrate	*GALE, GALK1, GALT*
	Others	*ABCD4, BTD, DNAJC19, GCH1, HCFC1, HSD17B10, MTHFR, MTR, MTRR, SLC52A1, SLC52A2, SLC52A3, SLC5A6, SPR*
**Mitochondrial disease**	OXPHOS system	*NDUFA2, NDUFA9, NDUFA10, NDUFA11, NDUFA12, NDUFA13, NDUFB3, NDUFB9, NDUFS1, NDUFS2, NDUFS3, NDUFS4, NDUFS6, NDUFS7, NDUFS8, NDUFA1, ACAD9, NDUFAF1, NDUFAF2, NDUFAF4, NDUFAF5, NDUFAF6, FOXRED1, SDHA-D, SDHAF1, SDHAF2, UQCRB, UQCRQ, BCS1L, TTC19, COX6B1, NDUFA4, COX4I2, SURF1, SCO1, SCO2, COX10, COX15, LRPPRC, COA5, ATP5E, ATPAF2, TMEM70, COQ2, COQ4, COQ6, PDSS2, ETFDH, ADCK3*
	Mitochondrial translation	*MRPS16, MRPS22, GFM1, TUFM, TSFM, PUS1, MTFMT, AARS2, DARS2, EARS2, GARS, MARS2, RARS2, SARS2, YARS2*
	Nucleotide maintenance	*TYMP, RRM2B, SLC25A4, TK2, MPV17, DGUOK, SUCLA2, SUCLG1, DNA2, SLC25A3, GFER*
	Others	*POLG, c10orf2, POLG2, OPA1, PINK1, DNM1L*
**Salt losing tubulopathy**		*BSND, CLCNKA, CLCNKB, KCNJ1, SLC12A1, SLC12A3*

PCCD: progressive cardiac conduction disturbance, CPVT: catecholaminergic polymorphic ventricular tachycardia, ARVC: arrhythmogenic right ventricular cardiomyopathy, OXPHOS: oxidative phosphorylation.

**Table 3:** Results of known arrhythmia-, inherited metabolic disease-, mitochondrial disease-, and salt-losing tubulopathy-related variants.

Case	Gene	Variant	Coordinate	Amino Acids	Zygosity	Heredity	Genetic phenotype	Inheritance	SIFT	PolyPhen-2	dbSNP ID
**1**	*KCNE1*	C > C/T	35,821,680	Asp85Asn	Heterozygote	Mother	Long QT syndrome	AD	Deleterious (0.03)	Benign (0.441)	rs1805128
	*CLCNKB*	T > T/C	16,373,054	Ile85Thr	Heterozygote	Father	Bartter syndrome type 3	AR	Deleterious (0)	Benign (0.097)	rs202202425
	*NDUFS4*	A > A/G	52,979,034	Arg171Gly	Heterozygote	Father	Mitochondrial complex I deficiency	AR	Deleterious (0)	Probably damaging (1)	rs200758718
**2**	*CAV3*	G > G/A	8,775,602	Val14Ile	Heterozygote	Father	Long QT syndrome	AD	Tolerated-low confidence (0.66)	Benign (0.002)	rs121909281
**3**	*SLC25A3*	C > C/G	98,991,805	Leu152Val	Heterozygote	Mother	Mitochondrial phosphate carrier deficiency	AR	tolerated (0.31)	Benign (0.122)	–
	*MTHFR*	G > G/A	11,854,077	Arg473Trp	Heterozygote	Father	Homocystinuria due to MTHFR deficiency	AR	Tolerated (0.38)	Possibly damaging (0.696)	rs750510348
**4**	*MYBPC3*	G > G/A	47,371,592	Arg160Trp	Heterozygote	Father	Cardiomyopathy	AD	Deleterious (0)	Probably damaging (0.998)	rs193068692
	*PDSS2*	A > A/C	107,475,829	Phe398Leu	Heterozygote	Mother	Coenzyme Q10 deficiency	AR	Tolerated (0.16)	Possibly damaging (0.632)	rs74325037
	*GARS*	G > G/A	30,673,468	Glu738Lys	Heterozygote	Mother	Neuronopathy (mitochondrial disease)	AR	Deleterious-low confidence (0.01)	Benign (0.175)	rs181251337
	*CPT1A*	A > A/C	68,529,142	Leu630Arg	Heterozygote	Mother	CPT deficiency type IA	AR	Tolerated (0.35)	Benign (0.024)	–
**5**	*AKAP9*	A > A/G	91,712,512	Gln2730Arg	Heterozygote	Father	Long QT syndrome	AD	–	Possibly damaging (0.474)	rs80191629
	*CACNA1C*	G > G/A	2,795,380	Arg1993Gln	Heterozygote	Mother	Long QT/Short QT syndrome	AD	–	–	rs190288386
	*ADCK3*	A > A/G	227,152,710	Lys63Glu	Heterozygote	Father	Coenzyme Q10 deficiency	AR	Tolerated-low confidence (0.27)	Benign (0.028)	–
	*TTC19*	T > T/G	15,902,833	Ile26Leu	Heterozygote	Father	Mitochondrial complex III deficiency	AR	tolerated (0.5)	Benign (0.002)	–
**7**	*CACNA1C*	G > G/A	2,795,380	Arg1993Gln	Heterozygote	Mother	Long QT/Short QT syndrome	AD	–	–	rs190288386
	*LRPPRC*	C > C/T	44,177,714	Glu559Lys	Heterozygote	Mother	Mitochondrial complex IV deficiency	AR	Tolerated (1)	Benign (0)	–
	*PUS1*	TG > TG/T	132,414,323	Gly20AspfsTer115	Heterozygote	Mother	Myopathy, Lactic acidosis	AR	–	–	–
	*PCCB*	A > A/G	136,046,480	Tyr455Cys	Heterozygote	Mother	Propionicacidemia	AR	Deleterious-low confidence (0)	Probably damaging (0.999)	rs121964961

MTHFR: methylenetetrahydrofolate reductase, CPT: carnitine palmitoyltransferase, AD: autosomal dominant, AR: autosomal recessive.

### Arrhythmia- and cardiomyopathy-related gene variants

Most cases of genetic arrhythmia have autosomal dominant (AD) inheritance, and a familial history is not always present. Sporadic cases are mostly caused by de novo variant^9^. Case 1 had Asp85Asn-*KCNE1*, AD inheritance of a long QT syndrome (LQTS)-related gene variant. This variant was reported to be associated with LQTS and suggested as a possible cause of death in SUDI^9,27^. The same variant was found in the female infant’s healthy living mother. Case 4 had Arg160Trp-*MYBPC3*, AD inheritance of a cardiomyopathy-related gene variant suspected to be harmful by in silico analysis. This variant reportedly could not be ruled out as a pathogenic variant^28^. The female infant’s father had the same variant but did not develop cardiomyopathy. Case 5 had Gln2730Arg-*AKAP9*, AD inheritance of an LQTS-related gene variant. *AKAP9* variants have been reported to be associated with LQTS type 11^29^, but Gln2730Arg-*AKAP9* has not previously been reported to be associated with LQTS type 11. The female infant’s living father had the same variant. Five of seven cases had synonymous variants that were inherited from either of the parents. In silico analysis showed that all of them were benign.

### Metabolic disease-, mitochondrial disease-, and salt-losing tubulopathy-related gene variants

Most inherited metabolic disease, mitochondrial disease, and salt-losing tubulopathy have autosomal recessive (AR) or X-chromosomal recessive inheritance. Homozygous amino acid changes or at least two heterozygous amino acid changes are necessary to cause these diseases^9,30–33^.

Case 1 had Leu21 = -*SDHAF2*, AD inheritance of a paraganglioma-related gene variant. TraP Score of Leu21 = -*SDHAF2* was 0.089, which meant that the variant was benign. In addition, her mother had the same variant. The diseases associated with the other 20 gene variants are all forms of AR inheritance. Since the variants in the cases were all heterozygous, these diseases were unlikely to have developed and led to death.

### Detected de novo, homozygous, compound heterozygous, and hemizygous variants aligned with all 4813 genes in TruSight One

A total of 12 de novo variants in 10 genes, 4 homozygous variants in 4 genes, compound heterozygous variants in 4 genes, and 4 hemizygous variants in 4 genes were found in < 0.1% of the Japanese population. All de novo variants were heterozygous (Table 4, Supplementary Table 2). As of August 2021, a search conducted on PubMed using these 20 gene names and the terms “sudden,” “infant,” and “death” as keywords returned no papers related to SUDI (Fig. 2).

**Table 4:** Results of de novo, homozygous, compound heterozygous, and hemizygous variants.

Case	Gene	Variant	Coordinate	Amino Acids	Zygosity	Heredity	Genetic phenotype	Inheritance	SIFT	PolyPhen-2	dbSNP ID	ToMMo frequency	HGVD frequency
**1**	*DRD5*	T > T/C	9,784,617	Cys322Arg	Heterozygote	De novo	Blepharospasm, ADHD	AD	Deleterious (0)	Probably damaging (1)	–	N.R	N.R
**2**	*COL5A1*	G > G/A	137,712,023	Arg1503His	Heterozygote	De novo	Ehlers–Danlos syndrome	AD	–	Unknown (0)	rs373653069	N.R	N.R
	*ZFHX3*	A > A/C	72,821,636	Ser3513Arg	Heterozygote	De novo	Prostate cancer	–	–	Unknown (0)	–	N.R	N.R
	*CD209*	C > C/T	7,810,559	Arg198Gln	Heterozygote	De novo	HIV type 1 susceptibility	–	Tolerated (1)	Benign (0.001)	rs41374747	N.R	N.R
	*CCKBR*	G > A/A	6,292,693	Asp422Asn	Homozygote	Both	pancreatic/gastric cancer	–	Tolerated (0.08)	Benign (0.002)	rs746523028	5/9544	2/2418
	*RGMA*	G > A/A	93,595,452	Ser147Leu	Homozygote	Both	Oral squamous cell carcinoma	–	Tolerated (0.25)	Probably Damaging (0.998)	rs200825597	8/9540	2/2418
	*DOCK6*	C > T/T	11,356,294	Arg323His	Homozygote	Both	Adams–Oliver syndrome 2	AR	Tolerated (0.05)	Probably damaging (0.911)	rs188066183	4/9546	2/2310
**3**	*OPTC*	T > T/G	203,466,112	Val80Gly	Heterozygote	De novo	High myopia	–	Tolerated (0.1)	Benign (0.013)	–	N.R	N.R
	*PRKDC*	G > G/A	48,771,471	Arg2095Cys	Heterozygote	De novo	Immunodeficiency	AR	–	–	rs8178147	N.R	N.R
**4**	*OBSCN*	G > G/A	228,412,409	Arg1060Gln	Heterozygote	Mother	Cardiomyopathy	–	–	Benign (0.037)	rs766325064	5/9546	N.R
	*OBSCN*	G > G/A	228,509,310	Ser5880Asn	Heterozygote	Father	Cardiomyopathy	–	–	Probably damaging (0.997)	rs765727329	3/9546	N.R
**5**	*KIR2DL4*	G > G/A	55,316,398	Arg76Lys	Heterozygote	Father	–	–	–	–	–	N.R	N.R
	*KIR2DL4*	C > C/A	55,317,489	Gln149Lys	Heterozygote	Ambiguous	–	–	–	–	rs796139593	N.R	N.R
	*KIR2DL4*	A > A/G	55,317,490	Gln149Arg	Heterozygote	Ambiguous	–	–	–	–	rs746343340	N.R	N.R
	*KIR2DL4*	G > G/A	55,317,528	Glu162Lys	Heterozygote	Ambiguous	–	–	–	–	rs540514355	N.R	N.R
	*KIR2DL4*	A > A/G	55,317,529	Glu162Gly	Heterozygote	Ambiguous	–	–	–	–	rs796093143	N.R	N.R
	*KIR2DL4*	T > T/C	55,317,541	Leu166Pro	Heterozygote	Ambiguous	–	–	–	–	rs112694450	N.R	N.R
	*KIR2DL4*	A > A/G	55,317,564	Ile174Val	Heterozygote	Ambiguous	–	–	–	–	rs200435373	N.R	N.R
**6**	*ZFHX3*	A > A/C	72,821,636	Ser3513Arg	Heterozygote	De novo	Prostate cancer	–	–	Unknown (0)	–	N.R	N.R
	*KMT5A*	T > T/C	123,892,078	Leu296Pro	Heterozygote	De novo	Malignant tumor of prostate	–	Deleterious (0)	Probably damaging (1)	–	N.R	N.R
**7**	*PIEZO2*	T > T/G	10,682,254	Lys2398Asn	Heterozygote	Father	Arthrogryposis, Marden–Walker syndrome	AD, AR	Deleterious (0)	Probably damaging (0.919)	–	N.R	N.R
	*PIEZO2*	T > T/C	10,770,217	Met934Val	Heterozygote	Mother	Arthrogryposis, Marden–Walker syndrome	AD, AR	Tolerated (1)	Benign (0)	–	2/9546	N.R
	*HCCS*	G > T/T	11,139,865	Ala248Ser	Hemizygote	Mother	Linear Skin Defects with Multiple Congenital Anomalies 1	XLD	Deleterious (0)	Probably damaging (0.996)	rs201692478	3/7412	2/2418
	*PHKA1*	A > G/G	71,830,918	Ile829Thr	Hemizygote	Mother	Muscle glycogenosis	XLR	Tolerated (0.6)	Benign (0.023)	–	N.R	N.R

ADHD: attention-deficit hyperactivity disorder, HIV: human immunodeficiency virus, AD: autosomal dominant, AR: autosomal recessive, XLD: X-linked dominant, XLR: X-linked recessive, N.R.: not reported.

## Discussion

First, we examined the significance of variants in disease-associated genes that could cause sudden death by performing trio analysis of SUDI cases and the parents. Arrhythmias, inherited metabolic disease, mitochondrial disease, and salt-losing tubulopathy were considered as candidate diseases that could cause SUDI since these diseases were difficult to diagnose because there are few specific findings at autopsy.

Arrhythmias and inherited metabolic disease have been reported to be associated with SUDI^3–10^. In this study, we adopted genes related to arrhythmia and inherited metabolic disease that we used in a previous study as candidate genes^9^. In particular, D85N_*KCNE1* has been previously reported as a genetic variant associated with LQTS. In the present study, the same variant was also found in the infant’s mother, which could suggest that this variant alone was not sufficient to cause LQTS.

Mitochondrial disease is a type of inherited metabolic disease that occurs in about 1 in 5000 births^34^. There have been reports of postmortem diagnosis of mitochondrial disease^11^. Ohtake et al. reported that about 9% of those diagnosed with mitochondrial disease were cases of SUDI^12^. In addition, we adopted this gene as a candidate because most mitochondrial diseases are caused by variants in human nuclear DNA^35^.

Salt-losing tubulopathy is a group of diseases, including Bartter syndrome and Gitelman syndrome, characterized by hypokalemic metabolic alkalosis as a common feature^36^. Electrolyte abnormalities cannot be established at general autopsy. In one case, Bartter syndrome was diagnosed by postmortem genetic testing^13^, and salt-losing tubulopathy-related genes were used as candidate genes.

After the filtering step, 34 variants were found aligned with the 31 target genes. All of these variants were heterozygous. Software Implemented Fault Tolerance (SIFT), PolyPhen2, and TraP Score revealed a total of nine harmful missense variants. Genetic analysis of this case alone would have determined that these variants were likely to cause abnormal protein function and were associated with cause of death. In particular, the *KCNE1*, *MYBPC3*, and *AKAP9* genes that were associated with LQTS or cardiomyopathy would have been suspicious as genes associated with cause of death because of an AD mode of inheritance^27,37,38^. These were sporadic cases without any family history of sudden death, and none of the AD-inherited arrhythmia-related gene variants in these cases were de novo. In general, if the proband had an AD genetic disease, at least some of the relatives would have the same symptoms. Therefore, these disease-associated genetic variants alone would not have caused the SUDI, as the relatives, including the parents, had not experienced SUD, obviously. These family histories and trio analysis results suggest that some of the variants in SUDI cases could not be involved in the development of the disease, although the same variants were categorized as pathological by in silico analysis or by the results of experimentation.

Second, candidate variants causing sudden infant death were extracted for all 4813 genes in the TruSight One panel. The incidence of SIDS in families is extremely low, and most cases are sporadic^39^. It is generally believed that rare variants are associated with rare diseases^40^, and diseases that can cause SUDI are considered to be one of the most severe of rare diseases. If SUDI cases with no family history of SUD manifested genetic variants that caused the sudden death, the variants were considered to be de novo. In addition, the variants of homozygotes or compound heterozygotes in SUDI cases and heterozygotes in their parents were extracted. Similarly, the variants of X-chromosome hemizygotes in SUD in male infancy and heterozygotes in their mothers were extracted.

We found eight de novo heterozygous missense variants in seven genes, three homozygous missense variants in three genes, eleven compound heterozygous missense variants in three genes, and two X-chromosome hemizygous missense variants in two genes (Table 4). These gene names and “sudden,” “death,” and “infant” were entered into PubMed as search terms. No papers described the association between these genes and SUDI. Therefore, some of these variants might be novel variants associated with cause of SUDI. Of these genes, we focused on two as biologically plausible candidate genes potentially associated with cause of death, *OBSCN* and *HCCS*.

Obscurin, which is encoded by the *OBSCN* gene, has an important role in the organization of sarcomeres during myofibril formation and the regular alignment of sarcoplasmic reticulum^41^. *OBSCN* variants may be monogenic causes of cardiomyopathy or contribute to the disease phenotype in concert with other variants^42^. In addition, inhibition of sarcomeric activity may cause arrhythmogenesis. It has also been reported that variants in the sarcomeric gene may have caused sudden cardiac death in a case of infant death without a cardiomyopathy phenotype^43^. In addition, the association of the *OBSCN* variant with a case of sudden cardiac death in an 8-year-old girl has also been reported^44^. Case 4 had maternal Arg1060Gln-*OBSCN* and paternal Ser5880Asn-*OBSCN* by compound heterozygotes. We speculated that these two heterozygous variants could be involved in sudden death by compound heterozygotes.

Holocytochrome c-type synthase, encoded by *HCCS* on the X chromosome, is located on the outer surface of the inner mitochondrial membrane and catalyzes covalent attachment of heme to both Cytc and Cytc1^45^. Cytc transfers electrons from electron-transfer complex III to complex IV in the mitochondria to promote ATP production. The *HCCS* variant at domain IV reduced expression of *HCCS* and also impaired release of Cytc^46^. These suggest that *HCCS* plays an important role in mitochondrial function. Mitochondrial dysfunction can lead to arrhythmia and sudden death^47^. In addition, it has been found that *HCCS* also has an important role in apoptosis and that the *HCCS* variant is associated with microphthalmia with linear skin defects syndrome, which is an X-linked male-lethal disorder^45,48^. These results suggest that *HCCS* is an essential protein for vital function. Case 7, a 6-month-old boy, had hemizygous Ala248Ser-*HCCS* in domain IV. The in silico algorithms predicted that it was deleterious and probably damaging, so we speculated that this variant was associated with the cause of death. However, there is no report about the clinical significance of Ala248Ser-*HCCS,* so a functional analysis study is needed.

Trio analysis was thought to be useful for both evaluation of disease-related gene variants that could cause sudden death and extraction of novel candidate genes for sudden death. Trio analysis revealed that some variants in existing candidate genes for cause of death might be insufficient to cause death because these variants were inherited from either of the parents. This means that the postmortem genetic analysis of only SUDI cases could have led to misdiagnosis and was considered insufficient to determine cause of death.

We extracted genes with de novo, homozygous, compound heterozygous, and hemizygous variants and reviewed previous reports that had reported an association between these genes and SUDI. There were no reports of associations between the genes found in this study and SUDI, which suggested that *OBSCN* and *HCCS* from these extracted genes are novel candidate genes associated with death caused by gene dysfunction or diseases related to genetic variants. In a large cohort study of whole-exome sequencing for SIDS, no variants were found to be significantly more prevalent in SIDS cases than in the general population^49^. Therefore, it may be difficult to identify gene variants that are candidates for cause of death by statistical analysis alone even when comparing with a large sample of unrelated healthy subjects. If a sudden death case has an ultra-rare variant, it could be possible to determine the variant with pathological significance by examining whether the parents also have the variant.

Trio-targeted gene-sequencing panel analysis has an advantage over conventional methods for determining the cause of death. Genetic analysis of not only SUD deceased infants but also living parents can be a more exact inquiry for cause of death by SUDI. An autopsy is an investigation of the cause of death that is performed directly from a corpse. However, a genetic analysis of the SUD infants and their parents can provide more accurate interpretation of genetic variants than previously possible and help confirm a diagnosis of the cause of death. Furthermore, trio analysis can be performed in daily medical practice. It is important that the family members of those who have died suddenly should undergo standardized testing, including genetic analysis^50–52^. We were careful to obtain informed consent from the parents before trio analysis. Additionally, if a trio analysis reveals a hereditary disease, it is possible to refer the patient for genetic counseling. Asymptomatic siblings can be provided with important genetic information of the deceased, which may enable detection of diseases before they develop. Furthermore, diagnosed individuals can receive appropriate treatment. Trio analysis data may eventually be utilized for child death reviews.

There were several study limitations that should be considered. Targeted gene-sequencing panel analysis was performed using a panel of 4813 genes; thus, targeted gene-sequencing panel analysis of genes not included in this panel and of the whole-genome sequence was not performed. TruSight One is a 4813-gene-sequencing panel that covers a wide range of known disease-associated genes, so it is helpful in clinical diagnosis^53^. However, we believe that whole-exome analysis and whole-genome analysis are necessary to search for unknown genetic diseases. Additionally, mitochondrial genome sequencing was not performed. In the 4813 genes analyzed in this study, only limited variants, such as synonymous, missense, insertions, and deletions, were examined, and copy number polymorphisms were not analyzed. In this study, we investigated the association between the cause of death in SUD cases and arrhythmia and cardiomyopathy-related gene variants extracted by trio analysis. However, since incomplete penetrance diseases were identified in which the parents were asymptomatic and the child had a severe phenotype^54^, further genetic studies including not only the parents, but also other relatives, are necessary.

In this study, we showed that trio analysis enabled more accurate interpretation of the clinically significant gene variants in SUD infants, which could help prevent misdiagnosis and extract novel genetic variants associated with SUDI. Trio analysis is a novel method that can be useful for determining cause of death because it also analyzes genetic information from living parents or relatives as well as from deceased victims. In addition, trio analysis may enable investigations of the cause of death even in hospitals. Trio analysis can identify unknown genetic variants that may be related to cause of death and help prevent SUDI in the future.

## Methods

### Study design and participants

The SUD cases of infants autopsied at ≤ 2 years of age were selected between April 2013 and March 2017. Informed consent for whole-genome sequencing and trio analysis were obtained from parents. A comprehensive forensic investigation, including a thorough examination of the death scene, a review of the clinical history, and performance of an autopsy that included macroscopic and microscopic examinations and a toxicology examination, were performed in all cases.

### Extraction of genomic DNA and genetic analysis

As reported previously^9^, genomic DNA of SUDI deceased infants and their living parents was isolated from blood leukocytes and buccal mucosa, respectively, by using the QIAamp DNA Blood Mini Kit (Qiagen, Tokyo, Japan) in accordance with the manufacturer’s standard methods. A TruSight One sequencing panel (Illumina, San Diego, CA, USA) targeting 4813 disease-associated genes was used, and the sequencing was performed on an Illumina MiSeq (Illumina) (Supplementary Text 1).

### Filtering steps for extraction of variants

As reported previously^9^, the sequencing reads were mapped to the hg19 human reference genome sequence by using Variant Studio (version 3.0) software (Illumina). The variants with a low Q30 score or a read depth of < 30 were excluded. Copy number variation was not analyzed in this study. To identify putatively pathogenic variants, those with allele frequencies < 1% in East Asian ethnic subgroups were retained and listed by using data from the dbSNP (http://www.ncbi.nlm.nih.gov/projects/SNP), the 1000 Genomes Project (http://www.1000genomes.org), the NHLBI Exome Sequencing Project (http://evs.gs.washington.edu/EVS), the Exome Aggregation Consortium (http://exac.broadinstitute.org). In silico algorithms, SIFT (http://sift.jcvi.org), PolyPhen-2 (http://genetics.bwh.harvard.edu/pph2), and TraP Score (http://trap-score.org/index.jsp)^55^, were used to predict whether the detected variants would affect the function of each protein. For the synonymous variants, the pathogenicity of the variant was confirmed by ClinVar (https://www.ncbi.nlm.nih.gov/clinvar/).

### Detected variants aligned with the target 216 genes

As reported previously^9^, from the TruSight One panel targeting 4813 genes, we extracted sequence information from the 216 genes for inherited arrhythmia, cardiomyopathy, metabolic diseases, mitochondrial disease, and salt-losing tubulopathy: 19 Brugada Syndrome genes, 15 LQTS genes, 6 short QT syndrome genes, 6 progressive cardiac conduction disturbance genes, 5 catecholaminergic polymorphic ventricular tachycardia genes, 5 arrhythmogenic right ventricular cardiomyopathy genes, 24 other cardiac genes, 63 inherited metabolic disease genes (such as fatty acid oxidation, amino acid, and organic acid disorders), 81 mitochondrial disease genes^30^, and 6 salt-losing tubulopathy genes (such as Bartter syndrome and Gitelman syndrome)^31–33^. The gene list and criteria of gene selection are provided in Table 2. Single-nucleotide variations causing synonymous substitutions, non-synonymous substitutions, nonsense substitutions, and insertions/deletions occurring in the coding regions were retrieved.

### Extraction of de novo, homozygous, compound heterozygous, hemizygous variants of SUDI

After filtering steps, to extract < 0.1% of Japanese allele variant frequencies, the data from the Genome Cohort Study of Tohoku Medical Megabank Organization (ToMMo) (http://ijgvd.megabank.tohoku.ac.jp) and the Human Genetic Variation Database (HGVD) in the Human Genetic Variation Browser (http://www.genome.med.kyoto-u.ac.jp/SnpDB/index.html) were referred to. Trio analysis was a comparative examination of genetic variants in SUDI deceased infants and their living parents. De novo variants, found in the SUD infants but not in their parents, were extracted by trio analysis. In the SUDI cases, homozygous variants or compound heterozygous variants that were heterozygous in their parents were extracted. In addition, in boy cases, hemizygous variants in X chromosome were extracted. Of these extracted variants, allele frequencies ≥ 0.1% in the Japanese subgroup in ToMMo and HGVD were excluded. Especially for the compound heterozygous variants, both allele frequencies of < 0.1% each were adopted. Sudden infant death candidate gene names and the terms “sudden,” “infant,” and “death” were combined and searched in PubMed.

### Sanger sequencing

Sanger sequencing was performed to confirm the detected candidate variants (*OBSCN* and *HCCS*), as previously described. The polymerase chain reaction primers were designed by using Primer3 version 0.4.0 (https://bioinfo.ut.ee/primer3-0.4.0). (Supplementary Table 3.)

### Ethical approval

Written informed consent was obtained from the parents for the use of the samples in this study, which was approved by the ethics committees of the Nagasaki University Graduate School of Medicine (20170504-3, 20200801-2). This study was performed in accordance with the Declaration of Helsinki.

## Supplementary Information

Supplementary Information 1.

Supplementary Information 2.

Supplementary Information 3.

Supplementary Information 4.

## Data Availability

The main data supporting the findings of this study are available within the article and its Supplementary Information. Additional data are available from the corresponding author upon reasonable request.
