# Supplementary material for: Accurate interpretation of genetic variants in sudden unexpected death in infancy by trio-targeted gene-sequencing panel analysis
Source: Sci Rep. 2021 Nov 2;11:21532. doi: 10.1038/s41598-021-00962-8 (PMC8563990; doi:10.1038/s41598-021-00962-8)
Supplement: Supplementary file 1 — Supplementary Information 1. [file 41598_2021_962_MOESM1_ESM.docx]

**Accurate interpretation of genetic variants in sudden unexpected death in infancy by trio-targeted gene-sequencing panel analysis**

Keita hingu^1,2^, Takehiko Murase^1^, Takuma Yamamoto^1,*^, Yuki Abe^1^, Yoriko Shinba^1^, Masahide Mitsuma^1,2^, Takahiro Umehara^1^, Hiromi Yamashita^3^, Kazuya Ikematsu^1^

^1^Division of Forensic Pathology and Science, Department of Medical and Dental Sciences, Graduate School of Biomedical Sciences, School of Medicine, Nagasaki University, Nagasaki, Japan.

^2^Departments of Pediatrics, Nagasaki University Graduate School of Biomedical Sciences, Nagasaki, Japan.

^3^Division of Forensic Dental Science, Department of Medical and Dental Sciences, Graduate School of Biomedical Sciences, School of Medicine, Nagasaki University, Nagasaki, Japan.

*Present address: Department of Legal Medicine, Hyogo College of Medicine, 1-1 Mukogawa-cho, Nishinomiya, Hyogo 663-8501, Japan.

Correspondence and requests for materials should be addressed to T.Y. (email: tk-yamamoto@hyo-med.ac.jp), Tel: +8195-819-7076

**Supplementary Table 1. Known arrhythmia-, inherited metabolic disease-, mitochondrial disease-, and salt-losing tubulopathy-related synonymous gene variants.**

| Case | Gene | Variant | Coordinate | Amino Acid | Zygosity | Heredity | Genetic phenotype | Inheritance | dbSNP ID | | TraP Score | | ClinVar |
| --- | --- | --- | --- | --- | --- | --- | --- | --- | --- | --- | --- | --- | --- |
| **1** | *SDHAF2* | A>A/G | 61205123 | Leu21= | Heterozygote | mother | Paragangliomas | AD | rs191513932 | | 0.089 | | likely benign |
|  | *SLC12A3* | C>C/T | 56936357 | Asn940= | Heterozygote | ambiguous | Gitelman syndrome | AR | rs13306666 | | 0.025 | | Conflicting interpretations of pathogenicity |
|  | *COX10* | C>C/T | 14095519 | Ala303= | Heterozygote | father | Mitochondrial complex IV deficiency | ― | rs370260574 | | 0.003 | | Conflicting interpretations of pathogenicity |
| **2** | *CACNA1C, CACNA1C-AS1* | C>C/T | 2788732 | Gly1786= | Heterozygote | mother | Long QT syndrome | ― | rs199538058 | | 0.145 | | benign/likely benign |
| **3** | *KCND3* | C>C/A | 112524479 | Arg290= | Heterozygote | father | Brugada syndrome | AD | rs200894990 | | 0.048 | | benign |
|  | *ANK2* | C>C/A | 114239726 | Gly950= | Heterozygote | father | Long QT syndrome | AD | rs189563238 | | 0.012 | | benign/likely benign |
|  | *MYH7* | G>G/A | 23889192 | His1196= | Heterozygote | mother | Cardiomyopathy | AD | rs777394877 | | 0.01 | | likely benign |
| **4** | *MARS2* | G>G/C | 198571302 | Val391= | Heterozygote | father | Combined oxidative phosphorylation deficiency | AR | rs117988876 | | 0.007 | | benign |
|  | *NDUFA12* | G>G/A | 95387987 | Asn72= | Heterozygote | mother | Mitochondrial complex I deficiency | AR | rs146044204 | | 0.026 | | benign |
|  | *POLG2* | A>A/G | 62474066 | Thr444= | Heterozygote | father | Mitochondrial DNA depletion syndrome | AR | rs782575432 | | 0.008 | | N.R. |
| **5** | *CLCNKA* | T>T/C | 16351310 | Leu94= | Heterozygote | mother | Bartter syndrome | DR | rs118025694 | | 0.091 | | N.R. |
|  | *SARS2* | G>G/A | 39410465 | Arg234= | Heterozygote | mother | Hyperuricemia | AR | rs35389151 | | 0.194 | | N.R. |
|  | *KCNE1* | G>G/A | 35821822 | Ser37= | Heterozygote | father | Long QT syndrome | AD | rs150458884 | | 0.096 | | Conflicting interpretations of pathogenicity |
| **6** | *KCNJ5* | G>G/A | 128786323 | Arg319= | Heterozygote | father | Long QT syndrome | AD | rs192889782 | | 0.138 | | benign/likely benign |
|  | *TSFM* | C>C/T | 58190150 | Arg275= | Heterozygote | mother | Combined oxidative phosphorylation deficiency | AR | ― | | 0.442 | | N.R. |
| **7** | *KCNQ1* | G>G/A | 2869008 | Leu602= | Heterozygote | mother | Long QT syndrome | AD | ― | | 0.179 | | likely benign |
| Abbreviations: AD: autosomal dominant, AR: autosomal recessive, DR: digenic recessive, N.R.: not reported.  ClinVar is a public archive of reports of the relationships among human genetic variations and phenotypes. | | | | | | | | | |  | |  | |
|  |  |  |  |  |  |  |  |  |  | |  | |  |
