# Supplementary material for: Accurate interpretation of genetic variants in sudden unexpected death in infancy by trio-targeted gene-sequencing panel analysis
Source: Sci Rep. 2021 Nov 2;11:21532. doi: 10.1038/s41598-021-00962-8 (PMC8563990; doi:10.1038/s41598-021-00962-8)
Supplement: Supplementary file 2 — Supplementary Information 2. [file 41598_2021_962_MOESM2_ESM.docx]

**Accurate interpretation of genetic variants in sudden unexpected death in infancy by trio-targeted gene-sequencing panel analysis**

Keita hingu^1,2^, Takehiko Murase^1^, Takuma Yamamoto^1,*^, Yuki Abe^1^, Yoriko Shinba^1^, Masahide Mitsuma^1,2^, Takahiro Umehara^1^, Hiromi Yamashita^3^, Kazuya Ikematsu^1^

^1^Division of Forensic Pathology and Science, Department of Medical and Dental Sciences, Graduate School of Biomedical Sciences, School of Medicine, Nagasaki University, Nagasaki, Japan.

^2^Departments of Pediatrics, Nagasaki University Graduate School of Biomedical Sciences, Nagasaki, Japan.

^3^Division of Forensic Dental Science, Department of Medical and Dental Sciences, Graduate School of Biomedical Sciences, School of Medicine, Nagasaki University, Nagasaki, Japan.

*Present address: Department of Legal Medicine, Hyogo College of Medicine, 1-1 Mukogawa-cho, Nishinomiya, Hyogo 663-8501, Japan.

Correspondence and requests for materials should be addressed to T.Y. (email: tk-yamamoto@hyo-med.ac.jp), Tel: +8195-819-7076

**Supplementary Table 2. *De novo*, homozygous, compound heterozygous, and hemizygous synonymous gene variants.**

| Case | Gene | Variant | Coordinate | Amino Acid | Zygosity | Heredity | Genetic phenotype | Inheritance | dbSNP ID | ToMMo frequency | HGVD frequency | Trap Score | ClinVar |
| --- | --- | --- | --- | --- | --- | --- | --- | --- | --- | --- | --- | --- | --- |
| **1** | *KRT5* | G>G/A | 52912783 | Arg239= | Heterozygote | *de novo* | Epidermolysis bullosa simplex | AD | ― | N.R. | N.R. | 0.099 | N.R. |
|  | *KRT5* | G>G/A | 52912786 | Gly238= | Heterozygote | *de novo* | Epidermolysis bullosa simplex | AD | ― | N.R. | N.R. | 0.075 | N.R. |
| **2** | *TRIP12* | C>T/T | 230668921 | Lys816= | Homozygote | both | Mental retardation | AD | rs776341102 | N.R. | N.R. | 0.239 | N.R. |
| **5** | *KIR2DL4* | C>C/A | 55317497 | Ser151= | Heterozygote | ambiguous | ― | ― | rs113248048 | N.R. | N.R. | 0.016 | N.R. |
|  | *KIR2DL4* | A>A/G | 55317530 | Glu162= | Heterozygote | ambiguous | ― | ― | rs796666015 | N.R. | N.R. | 0.089 | N.R. |
|  | *KIR2DL4* | C>C/T | 55317584 | Ala180= | Heterozygote | *de novo* | ― | ― | ― | N.R. | N.R. | 0.077 | N.R. |
| **6** | *KRT1* | G>G/A | 53070183 | Leu451= | Heterozygote | mother | Epidermolytic hyperkeratosis | AD | rs771675813 | N.R. | N.R. | 0 | N.R. |
|  | *KRT1* | T>T/A | 53073800 | Gly111= | Heterozygote | *de novo* | Epidermolytic hyperkeratosis | AD | ― | N.R. | N.R. | 0.088 | N.R. |
|  | *USP9X* | C>T/T | 41069798 | His1684= | Hemizygote | mother | Intellectual developmental disorder | XLD, XLR | ― | N.R. | N.R. | 0.061 | N.R. |
| **7** | *ALAS2* | G>A/A | 55042114 | Phe355= | Hemizygote | mother | Sideroblastic anemia | XLR | ― | N.R. | N.R. | 0.04 | N.R. |
| Abbreviations: AD: autosomal dominant, XLD: X-linked dominant, XLR: X-linked recessive, N.R.: not reported.  ClinVar is a public archive of reports of the relationships between human genetic variations and phenotypes. | | | | | | | | | | | | | |
