# Supplementary material for: Accurate interpretation of genetic variants in sudden unexpected death in infancy by trio-targeted gene-sequencing panel analysis
Source: Sci Rep. 2021 Nov 2;11:21532. doi: 10.1038/s41598-021-00962-8 (PMC8563990; doi:10.1038/s41598-021-00962-8)
Supplement: Supplementary file 3 — Supplementary Information 3. [file 41598_2021_962_MOESM3_ESM.docx]

**Accurate interpretation of genetic variants in sudden unexpected death in infancy by trio-targeted gene-sequencing panel analysis**

Keita hingu^1,2^, Takehiko Murase^1^, Takuma Yamamoto^1,*^, Yuki Abe^1^, Yoriko Shinba^1^, Masahide Mitsuma^1,2^, Takahiro Umehara^1^, Hiromi Yamashita^3^, Kazuya Ikematsu^1^

^1^Division of Forensic Pathology and Science, Department of Medical and Dental Sciences, Graduate School of Biomedical Sciences, School of Medicine, Nagasaki University, Nagasaki, Japan.

^2^Departments of Pediatrics, Nagasaki University Graduate School of Biomedical Sciences, Nagasaki, Japan.

^3^Division of Forensic Dental Science, Department of Medical and Dental Sciences, Graduate School of Biomedical Sciences, School of Medicine, Nagasaki University, Nagasaki, Japan.

*Present address: Department of Legal Medicine, Hyogo College of Medicine, 1-1 Mukogawa-cho, Nishinomiya, Hyogo 663-8501, Japan.

Correspondence and requests for materials should be addressed to T.Y. (email: tk-yamamoto@hyo-med.ac.jp), Tel: +8195-819-7076

**Supplementary Table 3. Primers for Sanger sequencing**

| Primer | Sequence |
| --- | --- |
| OBSCN_ Arg1060Gln_F1_outside | acagacagcccgtcatcagt |
| OBSCN_ Arg1060Gln_R1_outside | tgagccctgaccaaattctc |
| OBSCN_ Arg1060Gln_F2_inside | agcagcctctgcatctgaa |
| OBSCN_ Arg1060Gln_R2_inside | agcctacgctgaaacactgc |
| OBSCN_ Ser5880Asn _F1_outside | gctttttggaaggtgctgtc |
| OBSCN_ Ser5880Asn _R1_outside | ctcggcttcactctctgacc |
| OBSCN_ Ser5880Asn _F2_inside | aagggcatactggagcaaga |
| OBSCN_ Ser5880Asn _R2_inside | tgatcagcagagagcaggtg |
| HCCS_ Ala248Ser _F1_outside | tgataggcacgattggatca |
| HCCS_ Ala248Ser _R1_outside | gaggttgttcaggcttttcg |
| HCCS_ Ala248Ser _F2_inside | cgttgcgggacagaagttag |
| HCCS_ Ala248Ser _R2_inside | gcaattcaatctggggaaaa |
